# The optimal dietary arginine level of laying hens fed with low-protein diets

**DOI:** 10.1186/s40104-022-00719-x

**Published:** 2022-06-17

**Authors:** Mingfa Sun, Ning Ma, Hui Liu, Yu Liu, Yunlei Zhou, Jingpeng Zhao, Xiaojuan Wang, Haifang Li, Baishun Ma, Hongchao Jiao, Hai Lin

**Affiliations:** 1 Department of Animal Science, Shandong Agricultural University, Shandong Provincial Key Laboratory of Animal Biotechnology and Disease Control and Prevention, 61 Daizong Street, Taian City, 271018 Shandong Province China; 2 College of Chemistry and Material Science, Shandong Agricultural University, Taian City, 271018 Shandong Province China; 3 College of Life Sciences, Shandong Agricultural University, Taian City, 271018 Shandong Province China; 4 Shandong He-Mei-Hua Agricultural Technology Co., Ltd, Jinan City, 250101 Shandong Province China

**Keywords:** Anti-oxidation, Arginine, Laying performance, Low protein diet

## Abstract

**Background:**

Arginine (Arg) is an essential amino acid (EAA) in poultry, an important substrate for protein synthesis and a precursor of several molecules. Supplementation of EAAs with low protein (LP) diet increases the utilization efficiency of dietary crude protein (CP). However, if the EAA requirement is changed in hens fed a LP diet remains to be elucidated. The aim of the present study was to evaluate the optimal level of dietary Arg in the LP diet of hens. A total of 1350 Hy-Line Brown laying hens were randomly allocated to six dietary treatments: a basal diet (16% CP, positive control), or an isoenergetic LP diet (14% CP, 0.80% Arg) supplemented 0, 0.05%, 0.10%, 0.15%, and 0.20% L-Arg, corresponding to 0.80%, 0.85%, 0.90%, 0.95% and 1.00% dietary Arg, respectively.

**Results:**

The feed efficiency was decreased (*P* < 0.05) by 0.80% and 1.00% Arg-LP diets, compared to control. Within LP diets, dietary Arg level had significant quadratic effects (*P* < 0.05) on laying rate, egg mass, and feed efficiency. Compared to control, the plasma CAT activity or T-AOC content were decreased by 0.80% (*P* < 0.001). However, the hens offered 0.85% and 0.90% Arg-LP diets had higher CAT activity (*P* < 0.001) than 0.80% Arg-LP diet. In contrast, 1.00% Arg-LP group had the highest MDA and the lowest T-AOC content in plasma, liver, duodenal and jejunal mucosa (*P* < 0.05). Compared to control, the villus height was decreased by 0.80%, 0.95% and 1.00% Arg-LP diets, while the villus height to crypt depth (V/C) ratio was reduced by 0.95% and 1.00% Arg-LP diets in duodenum.

**Conclusion:**

The result demonstrates that LP diet (14% CP) deficient in Arg (0.80% Arg) result in augmented oxidative damage and impaired development of intestinal mucosa. According to the quadratic broken-line regression model, the optimal dietary arginine levels for Hy-Line Brown laying hens fed with low protein diet (14% CP) aged 33 to 40 weeks are 0.85%, 0.86%, and 0.86% to obtained the maximum laying rate, egg mass, and feed efficiency, respectively.

**Supplementary Information:**

The online version contains supplementary material available at 10.1186/s40104-022-00719-x.

## Background

Arginine (Arg) is one of the essential amino acid (EAA) for poultry, functioning as an important substrate for protein synthesis and the precursor of signal molecule nitric oxide (NO) [1, 2]. Arginine and/or its derivatives has been reported to enhance growth performance, nutrient transporters expression, increase antioxidant ability, reduce superoxide release, and ameliorate lipid peroxidation [3, 4]. In chicken and mammal, Arg/NO is involved in the regulation of muscular protein synthesis [5, 6]. Arginine supplementation is beneficial for the maintenance of the intestinal mucosal integrity by ameliorating inflammatory response and modulating gut microbiota in broiler chickens challenged with *S. typhimurium* [7]. Chickens fed diet deficient in Arg have decreased protein accretion, resulting in problems in growth, anti-oxidation, and immunity [8, 9]. In laying ducks, the graded levels of L-Arg supplementation (from 0 to 0.88%) to a basal diet containing 0.66% Arg increased egg weight, yolk color score, yolk percentage, and shell thickness [10].

Essential amino acid cannot be synthesized by chickens and must be supplied from diet. Therefore, a relative high-protein diet is needed to satisfy the requirement of EAA for growth and laying performance of chickens [11]. In practice, with the supplementation of EAA, feeding a low-protein (LP) diet could increase the utilization efficiency of dietary crude protein (CP) and reduce nitrogen excretion, without deteriorating production performance [12, 13]. The studies on the effect of LP diets fortified with AA on laying hen performance is inconsistent. In laying hens, 13% LP diet supplemented with EAAs has comparable laying performances with the 16% to 16.5% CP diets [14]. There are contrary reports indicate that LP diet negatively influences the laying performances [15, 16]. For example, feeding 4% lower in protein level diet supplemented with EAA deteriorated the performance of laying hens [17]. Our previous work indicated that LP diet (9.2% CP) supplemented with crystalline AA suppressing appetite and apo-lipoprotein synthesis in laying hens, which was associated with the decreased laying performances [18]. Recently, Parenteau et al. [19] reported that the hens fed LP diet with CP reduced by 2% unit points showed optimal performance responses at higher Ile:Lys (LP diet 82% to 84% vs. control 80%), implying the possibility of changed EAA requirement of hens fed LP diet. However, a recent study by Dao et al. [20] reported the lack of effect of L-Arg supplementation on laying performance in hens fed a 13% protein diet compared with 17% CP. Hence, the dietary EAA requirement in laying hens fed a LP diet remains to be elucidated.

The aim of the present study was to evaluate the optimal level of dietary Arg in the LP diet of hens. The laying performance, egg quality, and anti-oxidant capacity were evaluated. The expression of genes related to AA transporters and mucosa villus height and crypt depth in the small intestinal segments were also determined.

## Methods

### Birds and dietary treatment

A total of 1350 32-week-age Hy-Line Brown laying hens with similar body weight (BW, 2.02 ± 0.06 kg) were randomly allocated to six groups, each group had 15 replicates of 15 hens. The experimental hens were randomly subjected to one of the following dietary treatment: fed a basal diet (16% CP, positive control), or a isoenergetic low protein diet (14% CP, 0.80% Arg) supplemented 0, 0.05%, 0.10%, 0.15%, and 0.20% L-Arg, corresponding to 0.80%, 0.85%, 0.90%, 0.95% and 1.00% dietary Arg, respectively. Ingredient and nutrient composition of the experimental diets are shown in Table 1.

**Table 1:** Ingredient and nutrition composition of experimental diets (DM basis)

Ingredients, %	Control^a^	Dietary arginine level in LP diet, %^b^				
		0.80	0.85	0.90	0.95	1.00
Corn	62	68.61	68.55	68.51	68.46	68.41
Soybean meal (46% CP)	23	16.81	16.81	16.81	16.81	16.81
Soybean oil	1	–	–	–	–	–
Limestone	8	8	8	8	8	8
Premix^c^	6	6	6	6	6	6
L-Lysine-H_2_SO_4_ (70%)	–	0.2	0.2	0.2	0.2	0.2
DL-Met (99%)	–	0.03	0.03	0.03	0.03	0.03
L-Thr (99%)	–	0.11	0.11	0.11	0.11	0.11
L-Trp (99%)	–	0.04	0.04	0.04	0.04	0.04
L-Val (99%)	–	0.1	0.1	0.1	0.1	0.1
L-Ile (99%)	–	0.1	0.1	0.1	0.1	0.1
L-Arg (99%)	–	–	0.05	0.10	0.15	0.20
Calculated analysis						
ME, kcal/kg	2643	2643	2643	2643	2643	2643
Trp, %	0.21	0.21	0.21	0.21	0.21	0.21
Analyzed values^d^, %						
CP	15.93	14.14	13.98	14.18	14.08	14.08
Ca	3.52	3.49	3.46	3.50	3.60	3.51
AP	0.46	0.46	0.47	0.45	0.42	0.46
Lys	0.93	0.91	0.94	0.92	0.89	0.90
Met	0.43	0.41	0.40	0.43	0.38	0.41
Met+Cys	0.69	0.66	0.69	0.69	0.66	0.68
Thr	0.69	0.65	0.68	0.65	0.65	0.67
Gly	0.50	0.47	0.49	0.47	0.47	0.48
Val	0.73	0.72	0.74	0.71	0.71	0.73
Ile	0.63	0.58	0.61	0.60	0.59	0.62
Leu	1.26	1.19	1.20	1.18	1.20	1.21
Phe	0.66	0.65	0.66	0.67	0.67	0.66
Asp	1.10	1.09	1.15	1.11	1.11	1.12
Ser	0.62	0.59	0.61	0.61	0.59	0.62
Glu	2.43	2.37	2.34	2.36	2.36	2.35
Ala	0.67	0.65	0.67	0.66	0.66	0.65
His	0.47	0.45	0.43	0.43	0.43	0.44
Arg	0.96	0.80	0.86	0.91	0.95	1.02
Arg:Lys	103	88	91	99	107	113

^a^The control hens were fed a diet with 16% protein level;

^b^The experimental groups were provided a 14% protein diets (0.80% Arginine) supplemented with 0, 0.05%, 0.10%, 0.15%, and 0.20% L-Arg, respectively;

^c^The premix provide the follow quantities (per kilogram): vitamin A, 230 KIU; vitamin D_3_, 75 KIU; vitamin K, 86 mg; vitamin B_1_, 60 mg; vitamin B_2_, 150 mg; vitamin B_6_, 75 mg; vitamin E, 500 mg; niacin, 750 mg; pantothenic acid, 200 mg; biotin, 4 mg; iron, 1.5 g; selenium, 10 mg; copper, 300 g; zinc, 2.3 g; iodine, 20 mg; manganese, 2.4 g, Ca, 13.83%; AP, 3.33%; Lys, 2.17%; Met, 2.93%; salt, 5.67%;

^d^The amino acid content in this table refer to total amino acid levels

The experimental hens were reared in battery cage (60-cm length × 45-cm width × 50-cm height) and each hen had approximately 900 cm^2^ of floor space. Housing temperature and relative humidity were maintained at 23 ± 2 °C and 65 ± 5%, respectively. The photoperiod was 16 h light and 8 h dark. Each cage was equipped with 1 nipple drinker and a feeder. All hens had free access to feed and water throughout the experimental period. The experiment lasted 9 weeks, including a 1-week acclimation period and 8-week formal experimental period.

The BW of hens was recorded at the beginning and end of the experiment. Egg number and weight were recorded daily and feed intake was recorded weekly. Feed efficiency was calculated as grams of feed consumed per gram of egg mass produced. At the end of week 4 and 8, random samples of 6 eggs per replicate (total of 540 eggs) were collected and analyzed for egg quality.

At the end of week 4 and 8, one hen was randomly selected from each replicate (*n* = 15) after overnight feed withdrawal. A blood sample was drawn from the left-wing vein with 5-mL heparinized syringe. The blood sample was collected with ice-cold tube. Plasma samples were obtained after centrifugation at 3000 × *g* for 15 min at 4 °C and stored at − 20 °C for further analysis.

At the end of experiment, eight hens were randomly selected from each treatment. After overnight feed withdrawal, the bird was sacrificed by exsanguination [18]. The small intestine was dissected from the mesentery and immediately placed on ice. Intestinal segments (2.5 cm in length) of duodenum, jejunum, and ileum were obtained and fixed in 4% neutral buffered formalin for future histological analysis. The intestinal mucosa was obtained in duodenum, jejunum, and ileum, respectively. The mucosa samples were snap frozen in liquid nitrogen and then stored at − 80 °C for further analysis.

### Egg quality measurement

Egg length and egg width were measured using vernier caliper and the egg shape index was calculated by dividing the egg width by the egg length. Eggshell thickness was measured by averaging the three locations on the egg (air cell, equator, and sharp end) using an eggshell thickness tester (ETG-1061, Tokyo, Japan). Eggshell strength was measured using an egg force reader (EFG-0503, Tokyo, Japan). Yolk color, Haugh unit, and the height of albumen were measured using the egg quality analyzer (EMT-5200, Tokyo, Japan). Yolk and albumen were separated and weighed using a sensitive weighting balance, and their relative proportions (% egg weight) were determined.

### Diet analysis

The experimental diets were analyzed for dry matter (DM; method 930.15), crude protein (CP; method 990.03), calcium (Ca; method 984.01), and phosphorus (AP; method 965.17) of basal diet as described by AOAC International [21]. Dietary AA were determined by ion-exchange chromatography with postcolumn ninhydrin detection using a Hitachi L-8900 AA Analyzer (Tokyo, Japan) after acid hydrolysis with 6 mol/L HCl and reflux for 24 h. Methionine and cysteine were analyzed as Met sulfone and cysteic acid after cold performic acid oxidation overnight before hydrolysis.

### Plasma parameters and free amino acids

Plasma alanine aminotransferase (ALT), aspartate aminotransferase (AST), total protein (TP), urate, urea nitrogen (urea-N), glucose (GLU), triglyceride (TG) and total cholesterol (TCHO) were analyzed with commercial kits by using the Hitachi L-7020 fully automatic biochemical analyzer (Tokyo, Japan). The autoanalyzer has validated for avian plasma samples. A total free AA (TAA) assay kit (AA-1-W, Keming, Suzhou, China) was used to determine the content of TAA in plasma, according to the manufacturer’s instructions.

Plasma free AA concentrations were determined by ionexchange chromatography using a Hitachi L-8900 AA Analyzer (Tokyo, Japan) under physiological fluid analysis conditions. Frozen plasma samples (800 μL) were thawed at 4 °C and then deproteinized with 40 mg of sulfosalicylic acid. The sample was mixed by vortexing with an oscillator (Guohua Electric Appliance, Changzhou, China). After sitting for 4 h at 4 °C, the sample was then centrifuged at 12,000 × *g* for 30 min. The supernatant fluid was collected and passed through a filter (0.2 μm) before amino acid analysis.

### Nitric oxide, nitric oxide synthase and anti-oxidative parameter measurement

The NO concentration, induced NO synthase (iNOS) and total NO synthase (TNOS) in plasma were assessed using commercial kits (Jiancheng Bioengineering Institute, Nanjing, Jiangsu, China). The content of malondialdehyde (MDA), protein carbonyl, total anti-oxidant capacity (T-AOC), antioxidant enzymes including catalase (CAT), superoxide dismutase (SOD), and glutathione peroxidase (GSH-PX) in plasma, liver, and duodenum, jejunum, and ileum mucosa were determined by using commercial kits (Jiancheng Bioengineering Institute, Nanjing, Jiangsu, China). The protein concentration of supernatants was assayed using a BCA protein assay kit (Beyotime, Shanghai, China) according to manufacturer’s instructions.

### Intestinal histological analysis

Formalin-fixed duodenum, jejunum, and ileum samples were embedded in paraffin and cut into 4-μm serial sections. Two sections from each tissue sample were selected and stained with hematoxylin-eosin for identification. Ten well-oriented villi and their associated crypt were selected for each section, measured under a lightmicroscope (CK-40, Olympus, Tokyo, Japan) at 40× magnification and analyzed with an Image Analyzer (Lucia Software. Lucia, Za Drahou, Czechia). The 20 measurements were averaged to yield 1 value per laying hen. These procedures were conducted by an observer unaware of the dietary treatments to avoid bias.

### RNA isolation and RT-PCR analysis

Total RNA was extracted from duodenal, jejunal and ileal mucosa tissues (100 mg, frozen in liquid nitrogen) with the TRIZOL (Invitrogen, Carlsbad, California, USA). The RNA concentration was determined with agarose gel electrophoresis and a spectrophotometry (Eppendorf, Hamburg, Germany) detecting the UV absorbance ratio at 260 nm and 280 nm (A_260/280 _≈ 1.75–2.01). Then 1 μg RNA was reverse-transcribed to complementary DNA (cDNA) using DNase I (Invitrogen, Carlsbad, California, USA) according to the manufacturer’s protocol. Real-time PCR was performed using ABI Quant Studio 5 PCR machine (Applied Biosystems; Thermo, Waltham, MA, USA). Following the manufacturer’s protocol, the cDNA was amplified in a 20-μL PCR reaction system containing 0.2 μmol/L of each specific primer (Sangon, Shanghai, China) and the SYBR Green master mix (Roche, Basel, Switzerland). The primers were designed with Primer 6.0 software, and were based on published target sequences (Table 2). Primer against GAPDH was used as internal controls, and all of the mRNA values were normalized with the differences between individual samples. The relative expression of genes was compared with the control group using cycle threshold (Ct) values [22].

**Table 2:** Primers used for real-time quantitative PCR^a^

Gene^b^	Genebank accession no.	Orientation	Sequences (5′ to 3′)	Product size, bp
*CAT1*	NM_001145490.1	Forward	GCTCTATGGTGTTGGAGGG	192
		Reverse	AATAAGCCACAAAGCAGATGAG	
*b*^*0,+*^*AT*	NM_001199133.1	Forward	TGTGTTGCTCTCTAACTGGCTG	154
		Reverse	CCTCCTTTCTGTTGTCCTGTTC	
*y*^*+*^*LAT1*	XM_418326.5	Forward	TCCTGGTCATAGTTCCTCTCTACA	243
		Reverse	CAATGTCAAGGCAACCCTAC	
*rBAT*	XM_004935370.2	Forward	TTGGCTTGGCAAAGGAGTC	146
		Reverse	TCGGAATAGGCTGTGATGCT	
*B*^*0*^*AT*	XM_419056	Forward	AATGGGACAACAAGGCTCAG	125
		Reverse	CAAGATGAAGCAGGGGGATA	
*EAAT3*	XM_424930	Forward	ACCCTTTTGCCTTGGAAACT	122
		Reverse	TTGAGATGTTTGCGTGAAG	
*PepT1*	NM_204365	Forward	ACACGTTTGTTGCTCTGTGC	122
		Reverse	GACTGCCTGCCCAATTGTAT	
*GAPDH*	NM_204305.1	Forward	ACATGGCATCCAAGGAGTGAG	144
		Reverse	GGGGAGACAGAAGGGAACAGA	

^a^PCR = Polymerase chain reaction

^b^*CAT-1*, Cationic amino acid transporter-1; *b*^*0,+*^*AT*, b^0,+^ amino acid transporter; *y*^*+*^*LAT1*, y^+^L amino acid transporter-1; *rBAT*, Related to b^0,+^ amino acid transporter; *B*^*0*^*AT*, B^0^ neutral amino acid transporter; *EAAT3*, Acidic amino acids transporter; *PepT1*, Intestinal peptide transporter-1

### Statistical analysis

Before analysis, all data were examined for the homogeneity and normal distribution plots of variances among the treatments by using UNIVARIATE procedure. For the laying performance, egg quality, plasma biochemical indices, NO, NOS, anti-oxidative parameters, intestinal morphology, and mRNA levels of AA transporters, a one-way ANOVA model was used to estimate the main effect of dietary treatment (SAS version 8.1; SAS Institute Inc., Cary, NC, USA). When the main effect of the treatment was significant, the differences between means were assessed by Tukey’s multiple comparisons test. The orthogonal comparisons were applied for linear and quadratic effects of Arg in LP diets. *P* < 0.05 was considered to be statistically significant. Laying hen Arg requirement was calculated using quadratic polynomial and quadratic broken line models based on laying rate, egg mass and feed efficiency. The regression was analyzed by the NLIN procedure of SAS described by Robbins et al. [23]. Dietary Arg level as the independent variable in the quadratic polynomial and quadratic broken line models.

## Results

### Laying performance

Dietary treatment had no detectable influence (*P* > 0.05) on laying rate, egg weight, egg mass, and final BW (Table 3). In contrast, feed efficiency was decreased (*P* < 0.05) by 0.80% Arg-LP diet and 1.00% Arg-LP diet, compared to control. Within LP diet treatments, dietary Arg level had significant quadratic effects (*P* < 0.05) on laying rate, egg mass, and feed efficiency.

**Table 3:** Effect of the dietary arginine level in a low-protein diet on the laying performance^1^

Item	Control^2^	Dietary arginine level in LP diet, %^3^					*P*-value		
		0.80	0.85	0.90	0.95	1.00			
							Diet^4^	Linear^5^	Quadratic^5^
Final BW, kg	2.08 ± 0.03	2.03 ± 0.05	2.04 ± 0.07	1.97 ± 0.05	2.05 ± 0.06	1.94 ± 0.09	0.637	0.435	0.749
Laying rate, %	88.38 ± 1.38	86.80 ± 1.71	90.66 ± 1.21	89.07 ± 1.08	88.02 ± 1.23	86.47 ± 1.21	0.247	0.403	0.030
Egg weight, g	62.65 ± 0.29	61.92 ± 0.30	62.18 ± 0.28	62.51 ± 0.25	62.45 ± 0.38	62.21 ± 0.39	0.629	0.370	0.209
Egg mass, g/hen/d	55.35 ± 0.84	53.74 ± 1.10	56.34 ± 0.64	55.67 ± 0.70	54.97 ± 0.85	53.78 ± 0.83	0.183	0.602	0.012
Feed intake, g/d	133.36 ± 0.72	135.86 ± 1.01	137.64 ± 1.43	136.32 ± 0.90	136.83 ± 1.03	135.47 ± 0.82	0.073	0.605	0.224
Feed efficiency, g/g	2.42 ± 0.03^b^	2.54 ± 0.05^a^	2.44 ± 0.02^ab^	2.46 ± 0.03^ab^	2.50 ± 0.03^ab^	2.53 ± 0.04^a^	0.040	0.714	0.019

^1^Data were presented as the mean ± SEM (*n* = 15)

^2^The control hens were fed a diet with 16% protein level

^3^The experimental groups were provided a 14% protein diets (0.80% Arginine) supplemented with 0, 0.05%, 0.10%, 0.15%, and 0.20% L-Arg

^4^Diet = effect of all the dietary treatments

^5^Linear and quadratic = effect of dietary arginine from 0.80 to 1.00% treatments

^a,b^: Means with different superscripts within the same column differ significantly, *P* < 0.05

### Egg quality

After 4 weeks of treatment, the 0.80% and 0.90% Arg-LP diets had higher egg shape index than that of 0.85% and 1.00% Arg-LP treatments (*P* < 0.05, Table 4). Compared with control, yolk color score was increased in all the LP diets (*P* < 0.001). Egg weight, eggshell thickness, eggshell strength, albumen height, Haugh units, and percentages of egg components were not altered (*P* > 0.05) by dietary treatments.

**Table 4:** Effect of the dietary arginine level in a low-protein diet on egg quality^1^

Item	Control^2^	Dietary arginine level in LP diet, %^3^					*P*-value		
		0.80	0.85	0.90	0.95	1.00			
							Diet^4^	Linear^5^	Quadratic^5^
Week 4									
Egg weight, g	63.86 ± 0.49	63.71 ± 0.48	63.81 ± 0.44	63.38 ± 0.47	62.87 ± 0.45	63.18 ± 0.45	0.611	0.135	0.827
Egg shape index, %	78.57 ± 0.32^ab^	79.22 ± 0.50^a^	77.98 ± 0.24^b^	79.17 ± 0.23^a^	78.68 ± 0.27^ab^	78.11 ± 0.24^b^	0.027	0.131	0.775
Eggshell thickness, ×10^−2^ mm	33.82 ± 0.35	34.11 ± 0.28	33.70 ± 0.27	34.17 ± 0.26	33.77 ± 0.26	33.77 ± 0.26	0.775	0.451	0.951
Eggshell strength, kg/cm^2^	4.90 ± 0.04	4.85 ± 0.05	4.72 ± 0.07	4.82 ± 0.07	4.84 ± 0.06	4.91 ± 0.05	0.211	0.230	0.208
Albumen height, mm	6.41 ± 0.16	6.33 ± 0.13	6.19 ± 0.11	6.08 ± 0.21	6.01 ± 0.13	6.09 ± 0.08	0.302	0.156	0.357
Yolk color score	7.40 ± 0.18^c^	8.04 ± 0.09^ab^	7.96 ± 0.13^ab^	7.80 ± 0.11^b^	8.08 ± 0.13^ab^	8.25 ± 0.11^a^	< 0.001	0.134	0.029
Haugh units	77.93 ± 1.11	77.02 ± 1.18	75.68 ± 1.06	75.02 ± 1.86	74.85 ± 1.05	75.06 ± 0.54	0.360	0.232	0.445
Yolk, %	27.02 ± 0.25	27.56 ± 0.32	27.19 ± 0.21	27.54 ± 0.23	27.47 ± 0.28	27.85 ± 0.19	0.247	0.280	0.237
Albumen, %	62.25 ± 0.25	61.61 ± 0.34	62.11 ± 0.25	61.67 ± 0.23	61.63 ± 0.31	61.28 ± 0.21	0.124	0.189	0.205
Eggshell, %	10.72 ± 0.08	10.82 ± 0.08	10.70 ± 0.10	10.80 ± 0.05	10.90 ± 0.07	10.87 ± 0.06	0.327	0.176	0.416
Week 8									
Egg weight, g	63.09 ± 0.42	63.72 ± 0.33	63.56 ± 0.45	64.03 ± 0.49	64.36 ± 0.46	63.74 ± 0.45	0.443	0.526	0.506
Egg shape index, %	78.02 ± 0.27	78.85 ± 0.27	78.67 ± 0.22	78.99 ± 0.32	78.64 ± 0.29	78.78 ± 0.35	0.252	0.864	0.970
Eggshell thickness, ×10^−2^ mm	32.04 ± 0.41	32.02 ± 0.25	32.17 ± 0.35	32.87 ± 0.36	32.58 ± 0.49	33.23 ± 0.34	0.126	0.017	0.997
Eggshell strength, kg/cm^2^	4.33 ± 0.09	4.40 ± 0.09	4.37 ± 0.09	4.45 ± 0.08	4.40 ± 0.12	4.23 ± 0.10	0.697	0.332	0.260
Albumen height, mm	5.02 ± 0.04^e^	5.32 ± 0.07^de^	6.22 ± 0.17^a^	5.79 ± 0.14^b^	5.40 ± 0.06^cd^	5.65 ± 0.11^bc^	< 0.001	0.702	0.011
Yolk color score	7.37 ± 0.13^b^	7.97 ± 0.08^a^	7.74 ± 0.12^a^	7.70 ± 0.09^a^	7.65 ± 0.13^ab^	7.74 ± 0.08^a^	0.008	0.084	0.098
Haugh units	70.63 ± 0.74^bc^	69.29 ± 0.88^c^	76.66 ± 1.23^a^	73.31 ± 1.10^b^	69.98 ± 0.49^c^	72.32 ± 0.92^bc^	< 0.001	0.560	< 0.001
Yolk, %	26.62 ± 0.21	26.99 ± 0.19	26.85 ± 0.21	26.78 ± 0.23	26.72 ± 0.24	26.74 ± 0.21	0.878	0.326	0.676
Albumen, %	63.24 ± 0.21	62.77 ± 0.22	62.89 ± 0.26	62.97 ± 0.26	63.20 ± 0.31	63.08 ± 0.25	0.773	0.240	0.718
Eggshell, %	10.15 ± 0.10	10.24 ± 0.08	10.26 ± 0.12	10.25 ± 0.10	10.09 ± 0.11	10.19 ± 0.06	0.781	0.319	0.980

^1^Data were presented as the mean ± SEM (*n* = 15)

^2^The control hens were fed a diet with 16% protein level

^3^The experimental groups were provided a 14% protein diets (0.80% Arginine) supplemented with 0, 0.05%, 0.10%, 0.15%, and 0.20% L-Arg

^4^Diet = effect of all the dietary treatments

^5^Linear and quadratic = effect of dietary arginine from 0.80% to 1.00% treatments

^a-e^: Means with different superscripts within the same column differ significantly, *P* < 0.05

At week 8, dietary treatment had significant influence on albumen height (*P* < 0.001), Haugh unit (*P* < 0.001), and yolk color score (*P* < 0.01, Table 4). The LP diet treatments had higher yolk color score than control treatment (*P* < 0.001). The 0.85% Arg group had the highest albumen height and Haugh units compared to other treatment groups (*P* < 0.001). Egg weight, egg shape index, eggshell thickness, eggshell strength, and percentages of egg components were not altered (*P* > 0.05) by dietary treatments.

### Plasma biochemical indices

At week 4, hens offered 0.90% and 1.00% Arg-LP diets had significantly lower TP content than the control hens (*P* < 0.05, Table 5). Diet treatment had significant effect on urea-N and urate levels and the 0.95% and 1.00% Arg-LP groups had higher urea-N level compared to the other groups (*P* < 0.01). In contrast, the hens in 1.00% Arg-LP diet had higher urate level compared to control and 0.85%, 0.90% and 0.95% Arg-LP diets (*P* < 0.01). The ALT, AST, TAA, Glu, TG, and TCHO levels were not changed by dietary treatments (*P* > 0.05).

**Table 5:** Effect of the dietary arginine level in a low-protein diet on plasma biochemical indices^1^

Item	Control^2^	Dietary arginine level in LP diet, %^3^					*P*-value		
		0.80	0.85	0.90	0.95	1.00	Diet^4^	Linear^5^	Quadratic^5^
Week 4									
ALT, U/L	13.2 ± 0.8	14.8 ± 1.1	14.3 ± 0.9	13.7 ± 0.9	14.6 ± 0.6	12.8 ± 0.5	0.504	0.207	0.739
AST, U/L	196.9 ± 14.7	183.7 ± 14.0	192.9 ± 12.4	168.4 ± 15.8	196.2 ± 5.8	175.7 ± 4.1	0.473	0.579	0.935
TP, μmol/L	54.8 ± 2.0^a^	52.4 ± 2.0^abc^	52.9 ± 1.6^abc^	47.6 ± 1.7^bc^	54.0 ± 1.7^ab^	46.5 ± 3.1^c^	0.041	0.176	0.806
Urea-N, mmol/L	1.70 ± 0.15^b^	1.73 ± 0.06^b^	1.73 ± 0.18^b^	1.92 ± 0.14^b^	2.57 ± 0.18^a^	2.43 ± 0.16^a^	0.001	< 0.001	0.720
Urate, μmol/L	136.1 ± 15.9^b^	157.1 ± 11.9^ab^	120.1 ± 8.2^b^	106.2 ± 7.9^b^	128.9 ± 19.0^b^	189.4 ± 22.9^a^	0.007	0.030	< 0.001
TAA, μg/mL	604.0 ± 34.8	659.1 ± 30.4	618.9 ± 26.8	616.3 ± 23.3	648.5 ± 42.5	611.6 ± 44.0	0.841	0.484	0.707
GLU, mmol/L	12.3 ± 0.3	12.6 ± 0.5	12.1 ± 0.5	11.1 ± 0.5	12.0 ± 0.5	12.3 ± 0.4	0.261	0.637	0.060
TG, mmol/L	11.4 ± 1.9	12.0 ± 1.8	9.94 ± 1.54	7.05 ± 0.97	10.7 ± 1.54	9.74 ± 0.78	0.225	0.491	0.085
TCHO, mmol/L	2.97 ± 0.64	2.46 ± 0.34	1.88 ± 0.22	1.66 ± 0.16	2.17 ± 0.23	1.99 ± 0.17	0.106	0.458	0.057
Week 8									
ALT, U/L	13.1 ± 0.69	11.2 ± 0.96	11.0 ± 0.75	9.90 ± 0.41	10.5 ± 0.47	11.4 ± 0.65	0.056	0.969	0.142
AST, U/L	194.2 ± 8.6^a^	184.0 ± 10.3^ab^	171.4 ± 6.1^ab^	163.5 ± 5.2^b^	166.5 ± 4.7^b^	185.3 ± 6.7^ab^	0.031	0.925	0.010
TP, μmol/L	50.7 ± 1.5^b^	54.0 ± 0.8^ab^	54.6 ± 0.7^ab^	54.9 ± 1.4^ab^	54.0 ± 1.8^ab^	57.1 ± 1.5^a^	0.045	0.212	0.458
Urea-N, mmol/L	1.08 ± 0.08^b^	1.17 ± 0.13^ab^	0.99 ± 0.10^b^	1.30 ± 0.13^ab^	1.24 ± 0.23^ab^	1.54 ± 0.12^a^	0.001	0.043	0.302
Urate, μmol/L	122.0 ± 10.3^b^	117.2 ± 11.2^b^	126.0 ± 17.3^b^	176.2 ± 24.7^a^	142.1 ± 9.9^ab^	166.3 ± 18.2^ab^	0.048	0.031	0.355
TAA, μg/mL	698.3 ± 16.3	735.7 ± 20.1	766.4 ± 17.4	736.5 ± 26.1	748.8 ± 24.0	735.5 ± 23.1	0.368	0.779	0.589
GLU, mmol/L	11.8 ± 0.14^c^	11.7 ± 0.19^c^	12.1 ± 0.18^bc^	13.0 ± 0.34^a^	12.3 ± 0.23^abc^	12.8 ± 0.31^ab^	0.002	0.005	0.171
TG, mmol/L	7.89 ± 1.53	8.11 ± 1.24	7.42 ± 1.45	9.88 ± 1.40	9.51 ± 2.08	12.3 ± 1.47	0.265	0.046	0.580
TCHO, mmol/L	1.85 ± 0.24	2.02 ± 0.19	1.66 ± 0.20	2.02 ± 0.21	2.02 ± 0.25	2.43 ± 0.21	0.253	0.102	0.186

^1^Data were presented as the mean ± SD (*n* = 15). Alanine aminotransferase (ALT), Aspartate aminotransferase (AST), Total protein (TP), Urea, nitrogen (urea-N), urate, Total amino acids (TAA), glucose, Triglyceride (TG), Total cholesterol (TCHO)

^2^The control hens were fed a diet with 16% protein level

^3^The experimental groups were provided a 14% protein diets (0.80% Arginine) supplemented with 0, 0.05%, 0.10%, 0.15%, and 0.20% L-Arg

^4^Diet = effect of all the dietary treatments

^5^Linear and quadratic = effect of dietary arginine from 0.80% to 1.00% treatments

^a-c^: Means with different superscripts within the same column differ significantly, *P* < 0.05

After 8 weeks of treatment, plasma AST activity was lower (*P* < 0.05) in hens fed with 0.90% and 0.95% Arg-LP diets compared to control (Table 5). The 1.00% Arg-LP diet had higher TP and urea-N levels than that of control (*P* < 0.05). In contrast, the hens fed with 0.90% Arg-LP diet showed higher urate level (*P* < 0.05) compared to control, 0.80% and 0.85% Arg-LP diets. The hens fed 0.90% and 1.00% Arg-LP diets had higher glucose level (*P* < 0.05) than that in control, 0.80% and 0.85% Arg-LP diets (Table 5).

### Nitric oxide, nitric oxide synthase and anti-oxidative parameters in plasma

At week 4 and 8, the MDA content was significantly increased (*P* < 0.05) by 1.00% Arg-LP diet, compared with control (Table 6). Compared to control, the CAT activity was decreased (*P* < 0.001) by 0.80% and 1.00% Arg-LP diets at week 4 and by all the LP diets at week 8. However, the hens offered 0.85% and 0.90% Arg-LP diets had higher CAT activity (*P* < 0.001) than 0.80% Arg-LP diet at week 4. The hens of 0.80% Arg-LP diet had lower T-AOC compared to control at week 8. The NO level and activities of TNOS, iNOS, T-SOD, and GSH-PX were not changed by dietary treatments at week 4 and 8 (*P* > 0.05).

**Table 6:** Effect of the dietary arginine level in a low-protein diet on plasma anti-oxidative parameters^1^

Item	Control^2^	Dietary arginine level in LP diet, %^3^					*P*-value		
		0.80	0.85	0.90	0.95	1.00			
							Diet^4^	Linear^5^	Quadratic^5^
Week 4									
MDA, nmol/mL	5.85 ± 0.54^b^	5.67 ± 0.25^b^	5.80 ± 0.24^b^	6.00 ± 0.34^b^	6.38 ± 0.54^ab^	7.56 ± 0.48^a^	0.022	< 0.001	0.092
T-AOC, mmol/L	0.26 ± 0.03	0.24 ± 0.03	0.24 ± 0.02	0.20 ± 0.03	0.18 ± 0.04	0.25 ± 0.02	0.435	0.814	0.373
NO, μmol/L	1.45 ± 0.23	1.35 ± 0.29	1.62 ± 0.21	1.21 ± 0.19	1.60 ± 0.26	1.70 ± 0.22	0.259	0.396	0.312
TNOS, U/mL	29.8 ± 1.54	28.0 ± 0.95	30.0 ± 1.50	29.3 ± 2.70	30.7 ± 1.69	32.3 ± 1.53	0.626	0.087	0.840
iNOS, U/mL	11.6 ± 1.28	12.2 ± 1.06	11.4 ± 0.82	9.78 ± 0.77	11.9 ± 1.26	13.2 ± 1.26	0.403	0.063	< 0.001
CAT, U/mL	4.62 ± 0.44^ab^	1.97 ± 0.39^c^	4.28 ± 0.91^ab^	5.81 ± 0.81^a^	2.93 ± 0.44^bc^	1.30 ± 0.29^c^	< 0.001	0.169	< 0.001
T-SOD, U/mL	119.8 ± 1.4	121.0 ± 0.7	121.5 ± 0.5	122.0 ± 0.7	121.6 ± 0.5	121.4 ± 0.8	0.539	0.691	0.375
GSH-PX, IU	1794 ± 102	1861 ± 68	1697 ± 76	1861 ± 53	1895 ± 94	1886 ± 71	0.484	0.391	0.812
Week 8									
MDA, nmol/mL	4.73 ± 0.63^b^	5.13 ± 0.53^b^	5.05 ± 0.54^b^	5.99 ± 0.44^ab^	4.39 ± 0.51^b^	6.91 ± 0.48^a^	0.040	0.153	0.240
T-AOC, mmol/L	0.26 ± 0.02^a^	0.15 ± 0.03^b^	0.24 ± 0.05^ab^	0.22 ± 0.03^ab^	0.21 ± 0.02^ab^	0.17 ± 0.04^ab^	0.035	0.963	0.112
NO, μmol/L	5.51 ± 1.07	5.27 ± 0.97	7.60 ± 0.98	10.44 ± 2.29	7.78 ± 1.92	5.25 ± 0.61	0.226	0.975	0.009
TNOS, U/mL	38.8 ± 1.45	39.1 ± 0.78	38.5 ± 0.88	38.9 ± 1.48	33.3 ± 3.51	41.6 ± 1.24	0.075	0.974	0.110
iNOS, U/mL	9.19 ± 0.95	9.60 ± 1.20	9.43 ± 0.41	9.06 ± 1.42	9.28 ± 1.87	9.87 ± 0.70	0.997	0.928	0.680
CAT, U/mL	7.23 ± 1.62^a^	3.21 ± 0.48^b^	3.79 ± 0.72^b^	3.18 ± 0.42^b^	2.98 ± 0.50^b^	2.45 ± 0.48^b^	0.002	0.161	0.325
T-SOD, U/mL	149.0 ± 4.8	137.7 ± 3.5	138.5 ± 4.2	145.4 ± 4.4	139.4 ± 4.8	143.9 ± 3.7	0.370	0.318	0.665
GSH-PX, IU	2809 ± 149	3130 ± 212	2980 ± 149	2777 ± 118	2684 ± 96	2888 ± 82	0.306	0.124	0.198

^1^Data were presented as the mean ± SD (*n* = 15). Malondialdehyde (MDA) contents, Total antioxidant capacity (T-AOC), Nitric oxide (NO), Total nitric oxide synthase (tNOS) enzyme activity, Inducible nitric oxide synthase (iNOS) enzyme activity, Catalase (CAT) activity, Total superoxide dismutase (T-SOD) activity, Glutathione peroxidase (GSH-PX)

^2^The control hens were fed a diet with 16% protein level

^3^The experimental groups were provided a 14% protein diets (0.80% Arginine) supplemented with 0, 0.05%, 0.10%, 0.15%, and 0.20% L-Arg

^4^Diet = effect of all the dietary treatments

^5^Linear and quadratic = effect of dietary arginine from 0.80% to 1.00% treatments

^a-c^: Means with different superscripts within the same column differ significantly, *P* < 0.05

### Plasma free amino acids

The 0.85% Arg-LP diet had higher His and Ser levels compared to control (*P* < 0.05, Table 7). Dietary treatment, however, had no detectable effects on total indispensable AA, total dispensable AA and total AA contents (*P* > 0.05).

**Table 7:** Effect of the dietary arginine level in a low-protein diet on plasma free AA concentrations^1^

Amino acids, μg/mL	Control^2^	Dietary arginine level in LP diet, %^3^					*P*-value		
		0.80	0.85	0.90	0.95	1.00	Diet^4^	Linear^5^	Quadratic^5^
Indispensable AA									
Lys	63.69 ± 2.26	69.83 ± 5.02	79.15 ± 6.69	53.35 ± 6.69	66.08 ± 5.40	64.15 ± 4.53	0.075	0.286	0.500
Met	9.65 ± 0.44	9.45 ± 0.80	10.53 ± 0.57	10.36 ± 0.83	9.82 ± 0.57	10.91 ± 0.44	0.578	0.385	0.722
Arg	43.26 ± 1.74	42.91 ± 2.54	41.19 ± 3.01	50.57 ± 4.16	48.43 ± 2.00	44.61 ± 2.52	0.156	0.333	0.142
Val	3.35 ± 0.70	2.97 ± 0.30	3.64 ± 0.36	2.11 ± 0.23	2.33 ± 0.29	2.88 ± 0.26	0.070	0.145	0.209
Ile	9.83 ± 0.59	10.47 ± 0.75	10.87 ± 0.46	9.95 ± 0.86	9.86 ± 0.44	10.35 ± 0.70	0.836	0.562	0.708
Leu	19.60 ± 1.02	20.88 ± 1.11	21.87 ± 0.54	23.21 ± 2.26	20.62 ± 0.78	21.08 ± 0.92	0.440	0.797	0.289
Phe	13.18 ± 0.52	13.13 ± 0.35	13.58 ± 0.64	13.43 ± 0.58	13.83 ± 0.55	13.43 ± 0.26	0.929	0.557	0.604
Thr	28.11 ± 3.53	28.17 ± 2.54	32.36 ± 3.12	25.60 ± 2.51	24.02 ± 2.90	28.04 ± 1.61	0.409	0.352	0.653
His	14.95 ± 1.69^b^	18.29 ± 0.94^b^	22.16 ± 0.87^a^	17.39 ± 0.99^b^	18.04 ± 1.15^b^	16.98 ± 1.39^b^	0.005	0.070	0.320
Dispensable AA									
Gly	33.41 ± 2.23	33.77 ± 1.46	38.16 ± 1.86	33.99 ± 1.88	33.95 ± 1.95	31.88 ± 1.12	0.272	0.135	0.160
Tau	37.81 ± 3.84	32.05 ± 2.64	34.15 ± 2.10	27.74 ± 2.53	34.43 ± 2.22	35.99 ± 3.28	0.195	0.353	0.224
Asp	3.50 ± 0.62	2.65 ± 0.28	3.41 ± 0.49	3.84 ± 0.95	2.64 ± 0.66	2.00 ± 0.28	0.306	0.213	0.045
Ser	57.62 ± 3.13^b^	63.40 ± 3.15^ab^	71.98 ± 2.96^a^	66.75 ± 3.34^ab^	59.02 ± 3.75^b^	58.29 ± 2.73^b^	0.017	0.046	0.116
Ala	35.86 ± 2.59	34.80 ± 1.84	38.21 ± 1.30	34.35 ± 2.69	37.54 ± 3.90	37.97 ± 3.07	0.853	0.575	0.950
Cit	1.50 ± 0.06	1.61 ± 0.06	1.58 ± 0.08	1.42 ± 0.06	1.59 ± 0.07	1.49 ± 0.06	0.256	0.352	0.395
Cys	38.21 ± 4.14	38.74 ± 2.68	39.66 ± 1.71	36.82 ± 3.75	37.11 ± 1.78	38.81 ± 1.36	0.979	0.737	0.664
Tyr	17.24 ± 0.65	18.12 ± 1.95	19.47 ± 1.16	15.76 ± 0.74	18.28 ± 1.18	18.25 ± 0.89	0.364	0.712	0.531
Orn	6.97 ± 0.63	5.83 ± 0.66	7.01 ± 0.74	9.72 ± 1.93	6.59 ± 0.51	8.95 ± 0.90	0.080	0.116	0.341
Indispensable AA	205.6 ± 7.0	216.1 ± 10.9	235.3 ± 11.2	206.0 ± 9.5	213.0 ± 9.2	218.1 ± 6.6	0.255	0.538	0.909
Dispensable AA	232.1 ± 7.9	231.0 ± 7.1	253.6 ± 4.1	230.4 ± 10.4	231.2 ± 12.1	233.6 ± 7.6	0.365	0.465	0.549
Total AA	437.7 ± 12.8	447.1 ± 14.3	489.0 ± 14.8	436.4 ± 17.3	444.2 ± 20.0	456.3 ± 10.8	0.176	0.492	0.857

^1^Data were presented as the mean ± SD (*n* = 8)

^2^The control hens were fed a diet with 16% protein level

^3^The experimental groups were provided a 14% protein diets (0.80% Arginine) supplemented with 0, 0.05%, 0.10%, 0.15%, and 0.20% L-Arg

^4^Diet = effect of all the dietary treatments

^5^Linear and quadratic = effect of dietary arginine from 0.80% to 1.00% treatments

^a,b^: Means with different superscripts within the same column differ significantly, *P* < 0.05

### Anti-oxidative parameter in liver, duodenal, jejunal and ileal mucosa tissues

In the liver, dietary treatment had no influence (*P* > 0.05) on MDA and protein carbonyl contents (Table 8). The T-AOC level, however, was significantly decreased (*P* < 0.05) in 0.95% and 1.00% Arg-LP diets, compared to control. In duodenum and jejunum, the 1.00% Arg-LP diet had the highest level of MDA and the lowest T-AOC content, compared to other dietary treatments (*P* < 0.05). In ileum, dietary treatment had no detectable effect (*P* > 0.05) on MDA, protein carbonyl, and T-AOC levels.

**Table 8:** Effect of the dietary arginine level in a low-protein diet on anti-oxidative parameters in tissues^1^

Item, nmol/mg prot	Control^2^	Dietary arginine level in LP diet, %^3^					*P*-value		
		0.80	0.85	0.90	0.95	1.00			
							Diet^4^	Linear^5^	Quadratic^5^
Liver									
MDA	0.33 ± 0.03	0.35 ± 0.05	0.38 ± 0.03	0.36 ± 0.03	0.36 ± 0.04	0.40 ± 0.03	0.772	0.428	0.836
Protein carbonyl	1.56 ± 0.07	1.55 ± 0.14	1.27 ± 0.04	1.32 ± 0.07	1.72 ± 0.22	1.31 ± 0.08	0.059	0.619	0.747
T-AOC	78.9 ± 4.31^a^	74.6 ± 3.39^ab^	73.5 ± 4.81^ab^	72.0 ± 4.09^abc^	65.5 ± 3.28^bc^	61.0 ± 2.30^c^	0.022	0.003	0.370
Duodenum									
MDA	1.08 ± 0.16^ab^	0.73 ± 0.07^b^	0.60 ± 0.11^b^	1.20 ± 0.23^ab^	1.25 ± 0.14^ab^	1.62 ± 0.42^a^	0.027	0.002	0.592
Protein carbonyl	1.91 ± 0.09	1.88 ± 0.44	1.96 ± 0.23	1.98 ± 0.18	2.12 ± 0.16	1.67 ± 0.18	0.885	0.814	0.347
T-AOC	169.5 ± 6.6^bcd^	188.4 ± 6.3^ab^	195.0 ± 10.8^a^	180.1 ± 6.5^abc^	164.7 ± 6.0^cd^	150.6 ± 5.7^d^	< 0.001	< 0.001	0.127
Jejunum									
MDA	0.27 ± 0.05^b^	0.34 ± 0.08^ab^	0.22 ± 0.03^b^	0.34 ± 0.03^ab^	0.37 ± 0.05^ab^	0.47 ± 0.07^a^	0.044	0.032	0.108
Protein carbonyl	2.15 ± 0.58	2.87 ± 0.55	1.61 ± 0.32	2.43 ± 0.68	1.55 ± 0.32	2.81 ± 0.58	0.347	0.733	0.163
T-AOC	289.0 ± 17.7^a^	274.4 ± 10.3^a^	275.0 ± 11.7^a^	262.6 ± 11.9^ab^	269.2 ± 19.8^ab^	222.1 ± 11.5^b^	0.007	0.021	0.176
Ileum									
MDA	0.56 ± 0.10	0.61 ± 0.13	0.43 ± 0.05	0.67 ± 0.10	0.60 ± 0.12	0.52 ± 0.06	0.603	0.924	0.837
Protein carbonyl	2.18 ± 0.29	1.91 ± 0.09	2.32 ± 0.20	2.48 ± 0.28	1.89 ± 0.21	2.31 ± 0.14	0.319	0.452	0.246
T-AOC	283.8 ± 11.5	290.7 ± 14.2	281.9 ± 5.2	270.7 ± 9.1	281.6 ± 4.2	255.9 ± 8.5	0.205	0.028	0.752

^1^Data were presented as the mean ± SD (*n* = 8). Malondialdehyde (MDA) contents, Total antioxidant capacity (T-AOC)

^2^The control hens were fed a diet with 16% protein level

^3^The experimental groups were provided a 14% protein diets (0.80% Arginine) supplemented with 0, 0.05%, 0.10%, 0.15%, and 0.20% L-Arg

^4^Diet = effect of all the dietary treatments

^5^Linear and quadratic = effect of dietary arginine from 0.80% to 1.00% treatments

^a-d^: Means with different superscripts within the same column differ significantly, *P* < 0.05

### Intestinal morphology

In duodenum, the villus height was significantly decreased in 0.80%, 0.95% and 1.00% Arg-LP diets, while the villus height to crypt depth (V/C) ratio was reduced by 0.95% and 1.00% Arg-LP diets, compared to control (*P* < 0.05, Table 9, Additional file 1). In jejunum, the villus height was significantly decreased in 0.95% and 1.00% Arg-LP diets, while the V/C ratio was reduced by 1.00% Arg-LP diet, compared to control (*P* < 0.05). In ileum, however, the villus height, crypt depth, and the V/C ratio were not influenced by dietary treatments (*P* > 0.05).

**Table 9:** Effect of the dietary arginine level in a low-protein diet on intestinal morphology^1^

Item	Control^2^	Dietary arginine level in LP diet, %^3^					*P*-value		
		0.80	0.85	0.90	0.95	1.00	Diet^4^	Linear^5^	Quadratic^5^
Duodenum									
Villus height, μm	1854 ± 84^a^	1612 ± 47^bc^	1735 ± 35^ab^	1723 ± 26^ab^	1577 ± 62^bc^	1498 ± 71^c^	0.001	0.011	0.003
Crypt depth, μm	275.9 ± 26.0	269.2 ± 18.1	248.7 ± 12.0	304.9 ± 19.8	293.6 ± 9.6	290.7 ± 11.8	0.255	0.069	0.561
Villus height:crypt depth	7.17 ± 0.71^a^	6.17 ± 0.42^ab^	7.04 ± 0.22^a^	5.83 ± 0.40^ab^	5.43 ± 0.32^b^	5.25 ± 0.39^b^	0.011	0.004	0.318
Jejunum									
Villus height, μm	1574 ± 74^a^	1493 ± 34^ab^	1646 ± 43^a^	1544 ± 53^ab^	1394 ± 65^bc^	1313 ± 47^c^	0.001	< 0.001	0.004
Crypt depth, μm	225.9 ± 5.0	257.5 ± 24.2	236.0 ± 12.7	247.4 ± 12.8	223.1 ± 13.0	232.2 ± 8.9	0.499	0.166	0.654
Villus height:crypt depth	7.01 ± 0.40^a^	6.08 ± 0.47^ab^	7.08 ± 0.36^a^	6.35 ± 0.37^ab^	6.30 ± 0.22^ab^	5.69 ± 0.23^b^	0.012	0.155	0.048
Ileum									
Villus height, μm	1022 ± 48	1012 ± 52	1015 ± 25	965.1 ± 55.1	987.9 ± 26.1	930.3 ± 26.4	0.592	0.156	0.755
Crypt depth, μm	157.3 ± 4.7	192.4 ± 14.4	176.7 ± 6.8	174.4 ± 10.1	159.9 ± 8.0	173.3 ± 10.4	0.140	0.096	0.236
Villus height:crypt depth	6.51 ± 0.29	5.53 ± 0.59	5.80 ± 0.25	5.65 ± 0.39	6.28 ± 0.34	5.51 ± 0.37	0.340	0.729	0.385

^1^Data were presented as the mean ± SD (*n* = 8)

^2^The control hens were fed a diet with 16% protein level

^3^The experimental groups were provided a 14% protein diets (0.80% Arginine) supplemented with 0, 0.05%, 0.10%, 0.15%, and 0.20% L-Arg

^4^Diet = effect of all the dietary treatments

^5^Linear and quadratic = effect of dietary arginine from 0.80% to 1.00% treatments

^a-c^: Means with different superscripts within the same column differ significantly, *P* < 0.05

### Amino acid transporters related gene expression

**Fig. 1:**
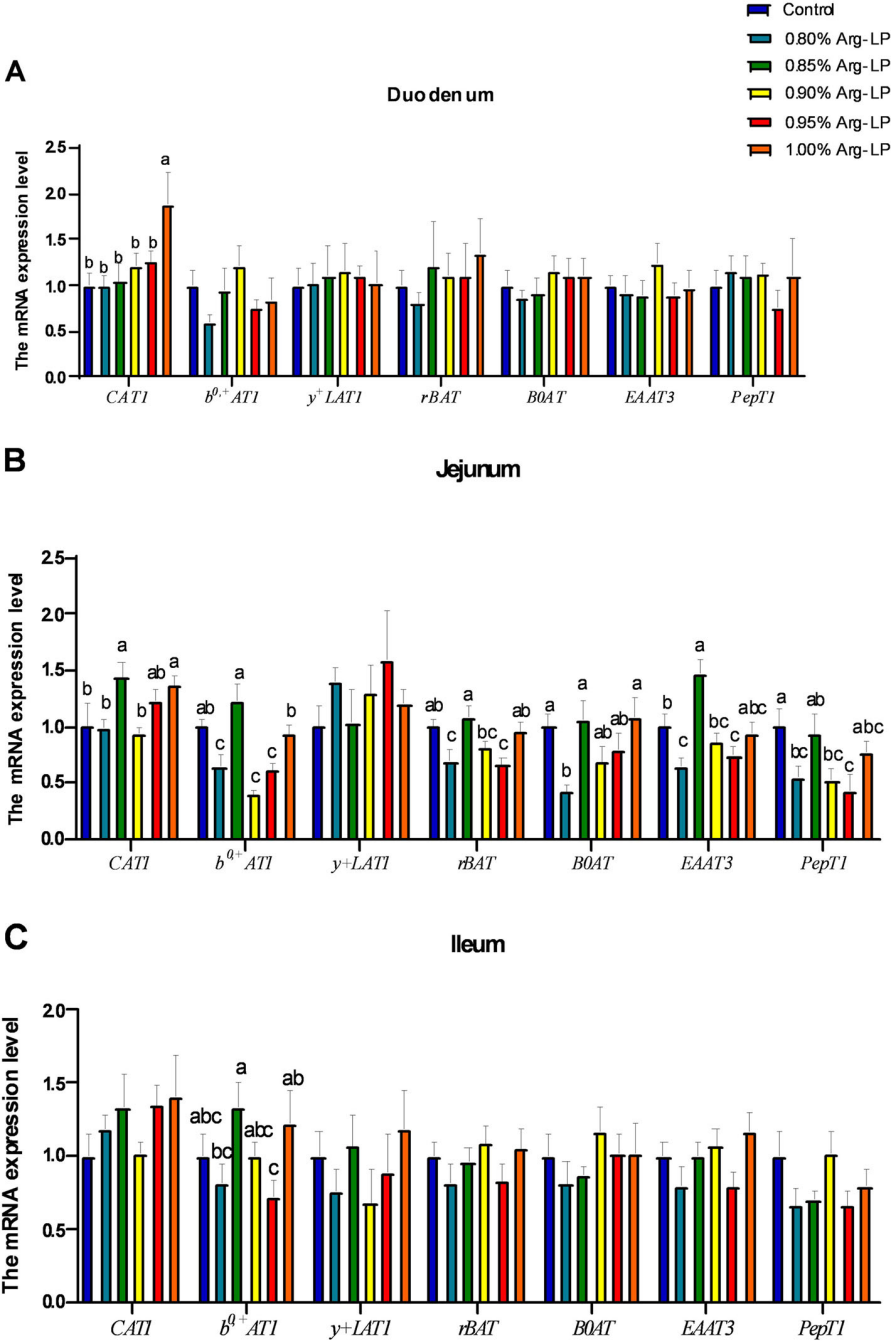
Effects of the dietary L-arginine level in a LP diet on the AA transporters expression. (**A**) Duodenum (**B**) Jejunum (**C**) Ileum. Data are shown as the mean ± SD (*n* = 8). ^a,c^: Means with different superscripts within the same column differ significantly, *P* < 0.05

In duodenum, *CAT1* expression was upregulated (*P* < 0.05, Fig. 1A) by 1.00% Arg-LP diet, while the expression levels of *b*^*0,+*^*AT1*, *y*^*+*^*LAT1*, *rBAT*, *B0AT*, *EAAT3*, and *PepT1* were not alted by dietary treatment (*P* > 0.05).

In jejunum, compared to control, 0.80% Arg-LP diet decreased the *b*^*0,+*^*AT1*, *rBAT*, *B0AT*, *EAAT3* and *PepT1* expressions (*P* < 0.05, Fig. 1B). However, the mRNA expression of *CAT1*, *b*^*0,+*^*AT*, *rBAT*, and *B0AT* was recovered by 0.85%-L-Arg diet or 1.00%-L-Arg diets (*P* < 0.05). The expression of *y*^*+*^*LAT1* was not changed by diteray treatment (*P* > 0.05).

In ileum, 0.85% Arg-LP diet significantly increased *b*^*0,+*^*AT1* expression compared with 0.80% and 0.95% Arg-LP diets (*P* < 0.05, Fig. 1C). In contrast, the expression of genes *CAT1*, *y*^*+*^*LAT1*, *rBAT*, *B0AT*, *EAAT3* and *PepT1* expression levels were not influenced by dietary treatments (*P* > 0.05).

## Discussion

These results demonstrate that LP diet (14%) not supplemented with L-Arg (0.80%) decreased the feed efficiency, reduced antioxidant capacity and suppressed duodenum villus height, which is alleviated by L-Arg supplementation (0.05–0.10%). However, when supplemented with higher than 0.95% Arg-LP diet, which made detrimental effects on the laying performance, anti-oxidant capacity and intestinal morphology.

The LP diet could increase the utilization efficiency of dietary CP and reduce nitrogen excretion, without deteriorating production performance [12, 13]. However, there are inconsistent reports in laying hens. The laying hens fed a 13% LP diet supplemented with EAAs has comparable laying performances with the 16% to 16.5% CP diets [14]. The present result showed that a reduction in the protein content of a laying hen’s diet from 16% to 14% resulted in reduced feed efficiency, in line with the work of Roberts et al. [15], who reported decreased egg production, egg mass and feed utilization efficiency when hens were fed an average of 19.34%, 17.38% and 16.13% vs. 20.10%, 18.44% and 17.28% during the 23 to 31, 32 to 44, and 45 to 58-week laying periods respectively. Similarly, decreasing dietary protein level from 16.18% to 14.16% had detrimental effects on the egg production and egg mass of layers between 28 to 40 weeks of age [16].

The balanced AA is important for the application of LP formulated to curtail production cost, NH_3_ emission and heat stress [24]. Arg, one of the EAA of chicken, plays an important role in the health, growth and egg production of hens. In the current study, the 0.80% Arg content in the LP diet without L-Arg supplementation was lower than the control diet (0.96%). The relative deficiency of Arg should be at least partially responsible for the decreased feed efficiency in LP diet-hens. This speculation was supported by the improved feed efficiency in LP diets supplemented with 0.05% and 0.10% L-Arg. In line with the result, Dao et al. [20] reported that the reduced egg weight by LP diet (13% CP vs. 17% CP) was corrected by the supplementation of L-Arg.

In Xinyang black laying hens (33 to 45 weeks), a local breed of China, fed with a 17% CP diet, the laying rate and feed efficiency showed a quadratic response to the dietary L-Arg levels, and 1.27% L-Arg group (Arg/Lys ratio = 187) had the highest laying rate and feed efficiency [25]. In Ross broiler breeders, dietary digestible Arg levels quadratically influenced the laying rate when fed with a 15.5% CP diet, and the highest laying rate was obtained when 1.262% Arg was fed [26]. The total Arg recommendation level for laying hens of 32 to 45 weeks of age is estimated at 760 mg/hen/d in the study by Leeson and Summers [27], while the digestible one is estimated to be 968 and 791 mg/hen/d for hens of 33 to 49 and 35 to 47 weeks of age [28]. In the present study, a quadratic effect of dietary Arg level in laying hens were observed on laying rate, egg mass and feed efficiency. According to the quadratic broken-line regression model, the optimal dietary total Arg level in LP diet was estimated at 0.85% (Arg/Lys ratio = 91), 0.86% and 0.86% (Arg/Lys ratio = 92) to obtained the laying rate, egg mass, and feed efficiency, respectively (Table 10). Consistent with our results, 0.68% Arg had higher laying rate and daily egg mass production than the 0.45% Arg group when supplemented with 11.9% CP diet in four genetically diverse purebred layer lines from 17 to 41 weeks [29]. However, Dao et al. [20] reported that the lack of effect of L-Arg supplementation (0.89% Arg; Arg/Lys ratio = 117) on laying performance in Hy-Line Brown hens when fed a 13% protein diet compared with 17% CP diet (0.90% Arg; Arg/Lys ratio = 118). The diet composition may influence Arg requirements. For example, the use of high level of corngluten in poultry diets may increase dietary levels of leucine. Therefore, leucine and lysine dietary levels should be considered in future study to determine Arg requirements more precisely [30]. Collectively, the present result suggest that the optimal Arg level is changed in hens fed with LP diet. In consideration of the possible influence of basal diet, breed and age of hens, further investigations are warranted.

**Table 10:** The optimal dietary arginine levels in laying hen fed with a low-protein diet

Criteria	Model	Independent variables	Regression equation	Arginine requirement	*R*^*2*^
Laying rate, %	Quadratic polynomial	Dietary Arg level	- 142.27 + 522.09*X* - 293.71*X*^2^	0.89%	0.73
	Quadratic broken-line	Dietary Arg level	90.67–1731.7(*X* - 0.85)^2^ - 27.24(*X* - 0.85)	0.85%	0.98
Egg mass, g/hen/d	Quadratic polynomial	Dietary Arg level	- 117.81 + 388.79*X* - 217.43*X*^2^	0.89%	0.82
	Quadratic broken-line	Dietary Arg level	56.45–686.7(*X* - 0.86)^2^ - 18.9(*X* - 0.86)	0.86%	0.97
Feed efficiency	Quadratic polynomial	Dietary Arg level	8.86–14.32*X* + 8.0*X*^2^	0.90%	0.77
	Quadratic broken-line	Dietary Arg level	2.44 + 26.43(*X* - 0.86)^2^ + 0.7(*X* - 0.86)	0.86%	0.96

From our findings, the yolk color score was lower in the control group than other treatment groups. Xanthophil is the major colorant responsible for the egg yolk color [31]. The increased yolk color score from hens fed LP diets maybe due to an increase in the consumption of corn-derived xanthophyils, since the LP diets contained a higher percentage of corn than the control diet in this research. This finding was in agreement with earlier reported researches [15, 32, 33]. Haugh units and albumen height are important parameters to evaluate the internal egg quality characteristics. Lieboldt et al. [29] reported that higher albumen proportion in hens fed Arg-sufficient LP diet (11.9% CP) compared to birds offered Arg-deficient LP diet. In the current study, 0.85% Arg-LP treatment had the highest Haugh units and albumen height compared with other treatment groups. Similar with our results, hens fed a diet with 1.27% L-Arg had the highest albumen height compared with that of other groups (0.64%, 0.86%, 1.03%, 1.42%, 1.66%) [25]. Arginine is the biological precursor of NO, which is associated with the regulation of protein synthesis in skeletal muscle of chickens [34]. Therefore, the effect of L-Arg and NO on the regulation of protein synthesis during egg formation and in turn the egg quality should be studied further.

The metabolism of L-Arg and other metabolites (ornithine and citrulline) in the ornithine cycle serves to dispose excess nitrogen by converting ammonia to urea. Arginase converts Arg into ornithine and urea-N. Kidney arginase activity is readily upregulated when excess Arg is provided in the diet [35]. It has been reported that between 40% to 60% of urea-N excreted by birds was from Arg metabolism [36]. In the present study, plasma urea-N and urate levels were increased by 0.95% and 1.00% Arg supplementation. In the present result, the unchanged L-Arg is related to the ornithine cycle, of which arginine is the component and is gently maintained within a relative stable level. Consistent with our results, Ruiz-Feria et al. [37] reported that when birds were fed high Arg levels, the plasma urea-N was higher than in Arg-water and medium-Arg feed. Meanwhile, urate is the main end product of nitrogen metabolism in birds. Plasma urate and urea-N can be used as indicators of AA utilization in broilers fed AA-adequate or AA-deficient diets [38]. In laying hens fed with a LP diet supplemented with all the EAAs, plasma urate was significantly increased simultaneous with the deteriorated laying performance [18]. Hence, the present result suggests that more than 0.95% L-Arg supplemental levels result in AA imbalance.

Oxidative stress is an important mechanism that leads to biological damage in living animals and causes several pathologies that affect poultry growth [39]. L-Arg is a substrate for NOS to produce NO [40]. Nitric oxide is a potent oxygen free radical scavenger induced by ochratoxin A in hepatocytes [41]. Arginine supplementation could increase NO bioavailability and reduce oxidative stress damage and improve the cardiovascular performance of broiler chickens grown under and chronic hypobaric hypoxia [42]. In broiler breeders in the late laying period, dietary supplementation of L-Arg could enhance the antioxidant capacity as well the laying performance [43]. In yellow-feathered chickens and laying ducks studies have shown that Arg decreased MDA levels in the serum, and enhanced the intestinal anti-oxidative defense system and reduced lipid peroxidation [44, 45]. In the present study, the hens fed with 0.80% Arg-LP diet had lower plasma CAT activity and T-AOC level but without change MDA and other antioxidant parameters at week 8, suggesting that LP diet has a minor effects on the antioxidant system of hens. In contrast, LP diet supplemented with 0.20% L-Arg showed increased MDA in plasma, duodenum, and jejunum, indicating that high supplemental level of L-Arg induces the augmented oxidative damage. The decreased T-AOC in plasma, liver, duodenum, and jejunum should be at least partially responsible for the oxidative stress. Indeed, NO may act as a free radical to form reacitve nitrogen specie (RNS) [46]. The RNS produced from NO includes many products such as nitrogen dioxide (NO_2_), dinitrogen trioxide (N_2_O_3_), nitroxyl anion (HNO), nitrosonium (NO^+^), nitronium (NO_2_^+^), peroxynitrite (ONOO^−^), etc. [47]. The uncontrolled production of ROS and RNS results in oxidative stress and causes damage in proteins, lipids, DNA, and cellular structures [48]. Hence, the present result suggestst that excessive supplementation of L-Arg in LP diet results in oxidative damage. It is interesting to note that plasma CAT activity was reduced but without altering SOD or GSH-PX activities at both week 4 and 8. This result was in line with the work by Delwing et al. [49], who reported that Arg decreased CAT activity and had no influence on SOD and GSH-PX in rat midbrain. L-NAME, an inhibitor of NOS, had no effect on CAT activity, suggesting that NO formation is involved in the reduction of CAT activity caused by L-Arg. The reduced CAT activity may contribute to the excessive L-Arg induced oxidative damage. The underlying mechanism needs to be investigated further.

Villus height and crypt depth are important factors that influence nutrient exchange area for digestion and absorption [50]. The villus height as well as the V/C ratio in the jejunum were decreased when dietary CP content was reduced from 18% to 14% or 16% CP [51]. In line with previous study, the present study indicated that the duodenal villus height was significantly decreased by 0.80% Arg-LP treatment, suggesting that LP diet impairs the proliferation and differentiation of enterocytes. Supplementation with Arg was shown to increase the intestinal concentration of polyamines and increased the cellular proliferation and intestinal repair after ischemia damage in rats [52]. Furthermore, Yuan et al. [53] demonstrated that Arg increased the proliferation of intestinal crypt cells from chicken embryos. In accordance with the previous works, 0.05% and 0.10% L-Arg supplementation (0.85% and 0.90% L-Arg-LP diets) partially recovered the detrimental effect of LP diet on villus height and the V/C ratio, suggesting that deficiency in Arg play a role in the deleterious influence of LP diet. In line with the result, a protective effect of Arg against LPS-induced enterocyte damage was observed in in vitro cultured porcine epithelial cells [54]. In contrast, the disadvantageous effect on intestinal morphology were observed in 0.95% and 1.00% Arg-LP diets indicated that excessive L-Arg level is not favorable for the development of intestinal tract. This result was supported by the observation of exaggerated oxidative damage in the intestinal tract. Nitric oxide is a free radical and weak oxidant. The bioavailability and actions of NO, however, are modulated by its fast reaction with superoxide radical, yielding an unusual and reactive peroxide ONOO^−^ [55]. The overproduction of NO or its toxic metabolite, ONOO^-^, promotes gut barrier failure [56]. ONOO^−^ may promote gut barrier failure not only by inducing enterocyte apoptosis but also by disrupting signaling pathways involved in enterocyte proliferation [57]. In this study, however, NO concentration and iNOS activity were not significantly changed by treatment. The role of NO in the oxidative stress induced by L-Arg supplementation should be explained with caution. Collectively, the result implies that LP diet increases the sensitivity of hens to Arg deficiency or overdose.

Dietary AA and peptides are absorbed via their specific transporters [12, 58, 59]. The expression of the cationic AA transporter b^0,+^ in the duodenum and CAT1 in the jejunum was influenced by dietary AA supplementation [60, 61]. The absorption of most AA occurs in the jejunum [62]. In the present study, the mRNA expression of jejunal AA transporters changed significantly with dietary treatments than other intestinal segments, suggesting that jejunal AA absorption is the site of most sensitive to Arg supplementation. Qiu et al. [51] reported that the expression of y^+^LAT1, rBAT, CAT1 and b^0,+^AT declined when dietary CP level was reduced from 18% to 14%. Consistent with our results, in jejunum, the mRNA expressions of genes *b*^*0,+*^*AT*, *rBAT*, *B0AT*, *EAAT3* and *PepT1* were decreased by LP diet without L-Arg supplementation when compared with control hens, revealing that LP diet had detrimental effects on the intestinal absorption. The unfavorable effect of LP diet on mRNA expression of *CAT1*, *b*^*0,+*^*AT*, *rBAT*, and *B0AT* was recovered by 0.05% (0.85%-L-Arg diet) or 0.20% (1.00%-L-Arg diet) L-Arg supplementation. It is well known that Arg regulates intestinal gene expression, growth and mucosal integrity, nutrient absorption, and metabolic pathways [63, 64]. The plasma free AAs, however, were not changed by LP or LP + L-Arg supplementation except of His and Ser, suggesting that adaptive balance of AAs. The plasma free AA pool comprises of the AAs absorbed from the digestive tract, mobilized from body protein, and uptake and clearance the move by tissues. Hence, the present result imply that the AA is balanced gently in hens fed a LP diet.

## Conclusion

According to the quadratic broken-line regression model, the optimal dietary arginine levels in LP diet for Hy-Line Brown laying hens aged 33 to 40 weeks are 0.85%, 0.86%, and 0.86% to obtained the maximum laying rate, egg mass, and feed efficiency, respectively. LP diet (14% CP) deficient in Arg (0.80% Arg) result in augmented oxidative damage and impaired development of intestinal mucosa.

## Supplementary Information

**Additional file 1 Fig. S1.** Effects of the dietary arginine level in a LP diet on the morphology of duodenum, jejunum, and ileum (40×, Scale bar = 500 μm)

## Data Availability

Datasets obtained and analyzed in this research are included within this article (and the supplementary data files).

## Abbreviations

ALT: Alanine aminotransferase
AP: Phosphorus
Arg: Arginine
AST: Aspartate aminotransferase
BW: Body weight
Ca: Calcium
CAT: Catalase
cDNA: Complementary DNA
CP: Crude protein
Ct: Cycle threshold
DM: Dry matter
EAA: Essential amino acid
GLU: Glucose
GSH-PX: Glutathione peroxidase
HNO: Nitroxyl anion
iNOS: Induced NO synthase
LP: Low protein
MDA: Malondialdehyde
NO: Nitric oxide
NO_2_: Nitrogen dioxide
N_2_O_3_: Dinitrogen trioxide
NO^+^: Nitrosonium
NO_2_^+^: Nitronium
ONOO^−^: Peroxynitrite
RNS: Reacitve nitrogen specie
SOD: Superoxide dismutase
TCHO: Total cholesterol
TP: Total protein
TG: Triglyceride
TNOS: Total NO synthase
T-AOC: Total anti-oxidant capacity
Urea-N: Urea nitrogen
V/C: Villus height to crypt depth
