# Supplementary material for: The optimal dietary arginine level of laying hens fed with low-protein diets
Source: J Anim Sci Biotechnol. 2022 Jun 17;13:63. doi: 10.1186/s40104-022-00719-x (PMC9206374; doi:10.1186/s40104-022-00719-x)
Supplement: Supplementary file 1 — Additional file 1 Fig. S1. Effects of the dietary arginine level in a LP diet on the morphology of duodenum, jejunum, and ileum (40×, Scale bar = 500 μm) [file 40104_2022_719_MOESM1_ESM.docx]

**Additional file 1** Effects of the dietary arginine level in a LP diet on the intestinal morphology

**1.00% Arg-LP**

**0.95% Arg-LP**

**0.90% Arg-LP**

**0.80% Arg-LP**

**0.85% Arg-LP**

**Control**

**Duodenum**

**
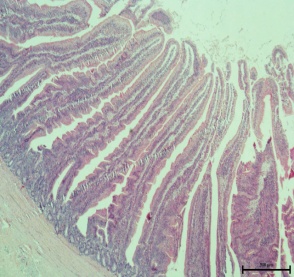

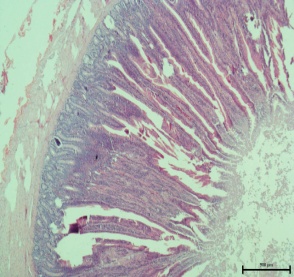

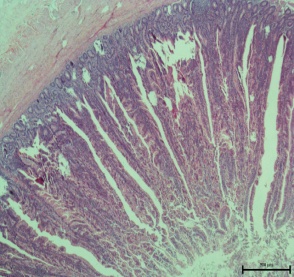

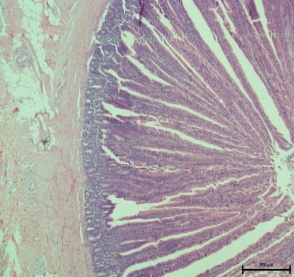

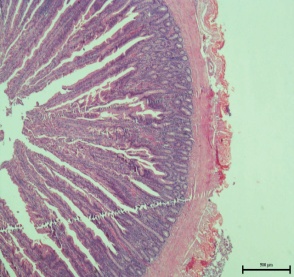

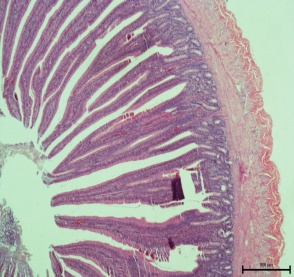
**

**Jejunum**

**
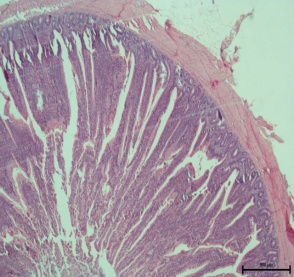

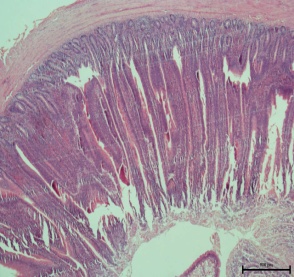

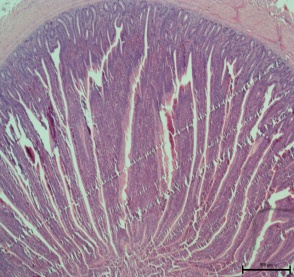

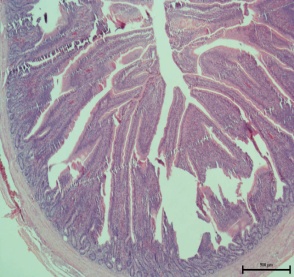

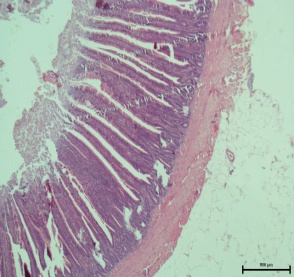

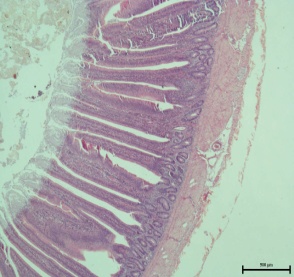
**

**Ileum**

**
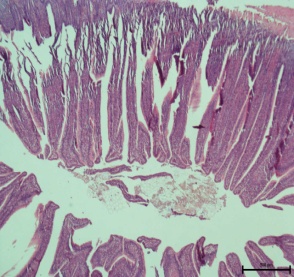

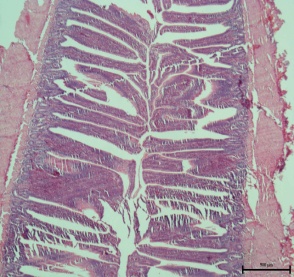

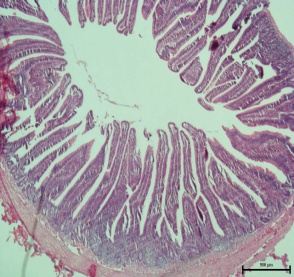

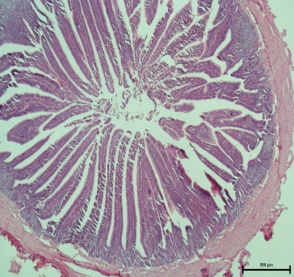

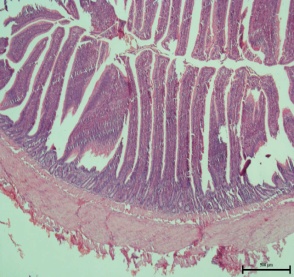

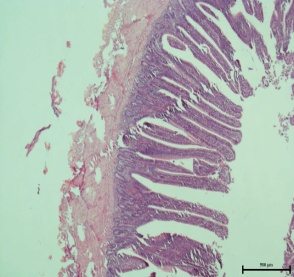
**
